# The Effect of Seated Elliptical Exercise on Chronic Low Back Pain: A Randomized Controlled Trial

**DOI:** 10.7759/cureus.99064

**Published:** 2025-12-12

**Authors:** Alireza Ebrahimi, Atta Taseh, Hannah McLeod, Kendal Toy, George R Malik, Alec L Meleger, Zacharia Isaac, Soheil Ashkani-Esfahani

**Affiliations:** 1 Department of Orthopaedic Surgery, Foot and Ankle Research and Innovation Laboratory (FARIL), Mass General Brigham, Harvard Medical School, Boston, USA; 2 Department of Physical Medicine and Rehabilitation, Mass General Brigham, Harvard Medical School, Boston, USA

**Keywords:** home-based exercise, low back pain, physical therapy rehabilitation, seated exercise, sedentary lifestyle

## Abstract

Background: Low back pain (LBP) is a prevalent health problem affecting a large portion of the population at some point in their lives, particularly with the increase in sedentary lifestyles, leading to significant disability and healthcare costs. This study evaluates the efficacy of seated elliptical exercise on chronic LBP, hypothesizing that daily use over a period of six weeks can reduce pain levels and improve functional health measures as measured by the Patient-Reported Outcomes Measurement Information System (PROMIS).

Methods: A randomized controlled trial involving 54 participants with chronic LBP was conducted, comparing a six-week seated elliptical exercise plan plus standard care against standard care alone. Outcome measures included the PROMIS Pain Intensity, Pain Interference, and Global Physical and Mental Health scores.

Results: The intervention group showed significant improvements in Pain Intensity (60.97±6.38 vs 57.41±6.98; P<0.001) and Pain Interference (62.61±7.10 vs 59.32±8.64; P<0.001), starting from the first week of exercise. While pain outcomes improved, no significant changes were observed in Global Physical and Mental Health measures.

Conclusion: Seated elliptical exercise might help reduce the pain in chronic LBP. This could be a promising intervention to relieve the pain or even prevent LBP, especially in individuals with a sedentary lifestyle.

## Introduction

The lifetime prevalence of low back pain (LBP) in the United States ranges from 60% to 70%, with approximately 20% of acute back pain cases progressing to chronic low back pain (CLBP), defined as LBP lasting for 12 weeks or longer [1]. Furthermore, LBP is a leading contributor to years lived with disability (YLDs) worldwide, imposing substantial direct costs related to healthcare utilization and indirect costs linked to lost productivity and disability [2]. Current management approaches emphasize conservative, non-invasive treatments such as physical therapy, pharmacological options, and psychological interventions [3]. Among these, exercise therapy plays a pivotal role, with well-documented benefits for pain reduction, functional improvement, and the prevention of symptom recurrence through targeted mobility enhancement [4]. 

Physical inactivity is a recognized risk factor for the onset and progression of CLBP [5]. Sedentary behaviors contribute to muscular deconditioning, spinal instability, and heightened susceptibility to symptom exacerbation [6]. Addressing these risks requires strategies aimed at promoting regular physical activity and breaking prolonged periods of inactivity. Interventions may include ergonomic adjustments, workplace modifications, and educational initiatives to incorporate movement into daily routines [7]. For individuals unable to participate in traditional outpatient programs, home-based training programs may offer an accessible alternative. These programs are designed to encourage gradual activity increases, focusing on restoring functionality and mitigating pain through low-impact exercises. Moreover, given the fact that a sedentary lifestyle might be inevitable due to the daily tasks and profession of some of these individuals, offering methods that can help increase their activity while sitting at the workplace or in between tasks can be an option to increase daily activity.

A previous report has demonstrated that seated elliptical exercise could mitigate sedentary behavioral habits [8]. Seated elliptical exercise, through engaging core and lower extremity muscles, could strengthen these muscles leading to lowering LBP. This exercise method could be an option for individuals with a sedentary lifestyle, who spend the majority of their working hours seated, and with restricted mobility, offering accessible and adaptable exercise. Additionally, this option is suitable for a wide variety of environments including outdoor, work, and home settings. The objective of this study is to investigate the effects of home-based seated elliptical exercise on patients with CLBP. We hypothesized that a six-week period of consistent use of this modality would reduce pain levels and improve functional outcomes measured by the Patient-Reported Outcomes Measurement Information System (PROMIS).

## Materials and methods

Study design and participants

This randomized controlled trial evaluated the efficacy of a home-based seated elliptical training plan as an adjunct to standard care, including physical therapy and analgesics, versus standard care alone in patients with CLBP. Participants were randomized to either the intervention group (seated elliptical plus standard method) or the control group (standard plan alone) using a computer-generated sequence with simple randomization. Randomization was conducted by an independent research staff member who had no role in outcome assessment. Blinding of participants and staff was not feasible due to the nature of the intervention.

Study participants were required to be adults aged 18-75 years with a documented history of CLBP persisting for 12 weeks or longer prior to enrollment. All participants were required to be able to provide informed consent and comply with study protocols. Individuals were excluded if they had experienced acute trauma within four weeks of enrollment, had confirmed spinal cord neoplasm, had active spinal infections, or had undergone spinal surgery within the previous six months. Additional exclusion criteria included current pregnancy, lower extremity pathology that would preclude the safe use of a seated elliptical device, and cardiovascular conditions that could limit exercise tolerance. 

Recruitment and consenting

**Figure 1:**
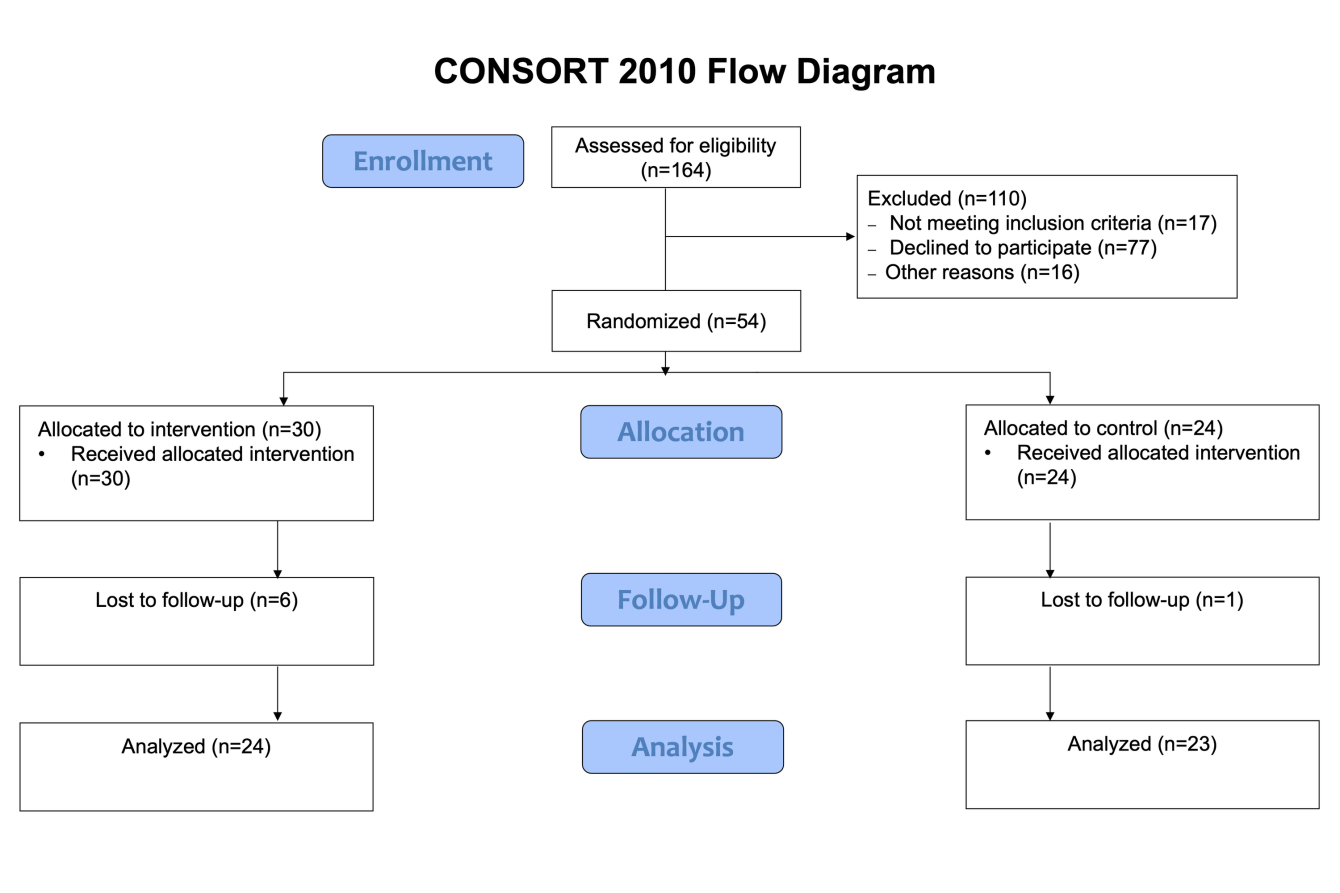
Study enrollment flow CONSORT: Consolidated Standards of Reporting Trials

After obtaining approval from the Mass General Brigham Institutional Review Board (approval number: 2022P001905), the study was conducted across four tertiary care centers, namely, Massachusetts General Hospital, Brigham and Women's Hospital, Newton-Wellesley Hospital, and Spaulding Rehabilitation Hospital, all located in Boston, Massachusetts. The study was also registered at ClinicalTrial.gov (ID: NCT05724160). Subject recruitment utilized two pathways: (1) direct referral by treating physicians during initial clinical encounters and (2) self-referral in response to recruitment advertisements in outpatient clinic settings. The latter method needed confirmation by an expert physician. Following the preliminary identification of eligible subjects, the process of consenting the individuals was started. Study procedures, including risks and benefits, were explained comprehensively. Informed consent was obtained from all participants. Participants were informed that their participation was voluntary and would not affect their standard medical care. The target sample size was 64 participants. However, recruitment was stopped early with 54 participants randomized (30 intervention, 24 control) due to funding limitations and a slower-than-expected recruitment rate. The study enrollment flow is presented in Figure 1. 

Intervention

Subjects in the intervention group were provided with a commercially available seated elliptical device (Cubii Total Body+, Fitness Cubed Inc., Chicago, IL, USA) for home-based training concurrent with the standard of care. The prescribed exercise regimen consisted of a six-week program, with one session per day, five days per week. Each session's duration had to be at least 15 minutes and not to exceed 30 minutes. Our team were following the participants using follow-up checklists that were sent to them on a weekly basis. Participants were required to document exercise parameters including the time and mileage shown on the device's monitor, as well as perceived exertion, and completion status (exercise completed/not completed). The control cohort received standard of care, which consisted of clinician-prescribed physical therapy and the use of analgesics. Because management was tailored to each patient's clinical condition, no single uniform treatment was applied.

Outcome metrics

The study outcomes were measured every week across the six-week study period, utilizing selected instruments from the PROMIS including the PROMIS v2.0 Pain Intensity 3a (primary outcome), PROMIS Short Form v1.1 Pain Interference 8a, and multiple domains from the PROMIS Global Health assessment, specifically examining general health, mental health, social activities/roles, and physical health components [1]. All PROMIS measures utilize standardized T-scores (mean=50; standard deviation (SD)=10) calibrated to the general US population, with higher scores indicating greater severity of the measured domain. All instruments were administered in their validated English versions.

Statistical analysis

The analysis was done utilizing IBM SPSS Statistics for Windows, V. 28.0 (IBM Corp., Armonk, NY, USA). To compare the baseline characteristics of the study groups, the chi-squared test was used for categorical data, and the independent t-test and Mann-Whitney U test were used for normally and non-normally distributed data, respectively. The PROMIS T-scores were analyzed using the analysis of variance (ANOVA) test. Post hoc analysis with Bonferroni correction was employed in case of a significant main effect, and P-values for pairwise comparisons were presented. Patients who failed to participate after the baseline or the first-week assessments were excluded from the ANOVA analysis but still included in comparing baseline measurements. Data imputation was used to compensate for the missing data for other participants. Data were presented as mean±SD, percentage (%), or median and interquartile range (IQR), where applicable. A P-value of 0.05 was considered significant. Although recruitment was stopped early, a post hoc power analysis based on the observed between-group difference indicated that with the final sample (n=47), the study had an 84% power to detect an effect size of 0.91 at a two-sided α of 0.05.

## Results

A total of 54 participants were enrolled in the study. Seven patients did not adhere to the study protocol. As a result, data from 47 participants (23 in the control group and 24 in the intervention group) were included in the final ANOVA analysis. There were no significant differences in the baseline demographic characteristics between the two groups (Table 1).

**Table 1:** Comparison of the baseline characteristics of the study groups *Independent t-test. **Chi-squared test. ^†^Mann-Whitney U test IQR: interquartile range

Characteristic	Intervention	Control	P-value
Age (mean±SD)	51.93±16.03	49.26±18.10	0.58^*^
Gender (female %)	60.9%	82.1%	0.13^**^
BMI (kg/m^2^)	28.8 (24.6, 30.4)	30.4 (28.65, 36.45)	0.07^†^
Race (White %)	84.6%	89.5%	0.66^**^
Activity level (sedentary %)	70%	47.8%	0.1^**^
Symptom chronicity (median (IQR); weeks)	52 (42.25, 56.75)	52 (24.5, 52)	0.22^†^

The outcome of our initial repeated measures ANOVA test encompassing both groups showed that PROMIS Pain Intensity scores changed significantly during time in further follow-ups (P<0.001); however, our post hoc test showed that these significant changes during follow-ups were in the intervention group which exhibited improvement starting from the first week (P=0.02 vs baseline), with sustained effects throughout the study (P<0.001 vs baseline). On the other hand, the changes in the control group were not statistically significant (Table 2). 

**Table 2:** PROMIS Pain Intensity and Pain Interference scores of the study groups across the study time points. Values are presented as mean±standard deviation ^a^Baseline vs first week. ^b^Baseline vs second week. ^c^Baseline vs third week. ^d^Baseline vs fourth week. ^e^Baseline vs fifth week. ^f^Baseline vs sixth week PROMIS: Patient-Reported Outcomes Measurement Information System

Outcome		Baseline	1st week	2nd week	3rd week	4th week	5th week	6th week	P-value
Pain Intensity	Intervention (n=24)	60.97±6.38	57.41±6.98	55.35±6.19	56.62±6.61	54.29±6.13	53.55±6.26	53.42±6.34	0.02^a^, <0.001^b^, <0.001^c^, <0.001^d^, <0.001^e^, <0.001^f^
	Control (n=23)	59.52±6.14	58.24±7.66	56.48±5.03	57.92±5.93	58.50±6.22	57.25±5.40	58.90±5.66	1^a^, 0.84^b^, 1^c^, 1^d^, 1^e^, 1^f^
	P-value	0.24	0.92	0.52	0.57	0.03	0.03	0.004	
Pain Interference	Intervention (n=24)	62.61±7.10	59.32±8.64	56.27±7.54	57.49±8.11	56.45±8.19	56.09±7.62	56.65±7.66	0.01^a^, <0.001^b^, <0.001^c^, <0.001^d^, <0.001^e^, <0.001^f^
	Control (n=23)	59.75±5.16	57.57±5.92	56.59±5.31	58.14±4.60	58.78±5.04	58.17±3.82	57.72±5.66	0.31^a^, 0.04^b^, 1^c^, 1^d^, 1^e^, 1^f^
	P-value	0.12	0.42	0.86	0.74	0.24	0.24	0.59	

Similarly, the PROMIS Pain Interference scores demonstrated a significant trend of improvements based on the initial repeated measures ANOVA outcomes (P<0.001). The intervention group results were significantly improved starting from the first week (P=0.01 vs baseline) throughout the study period (P<0.001 vs baseline in the next follow-up visits), whereas the control group only showed significant outcomes in the second week (P=0.04 vs baseline), which was not maintained in later follow-ups (Table 2).

**Figure 2:**
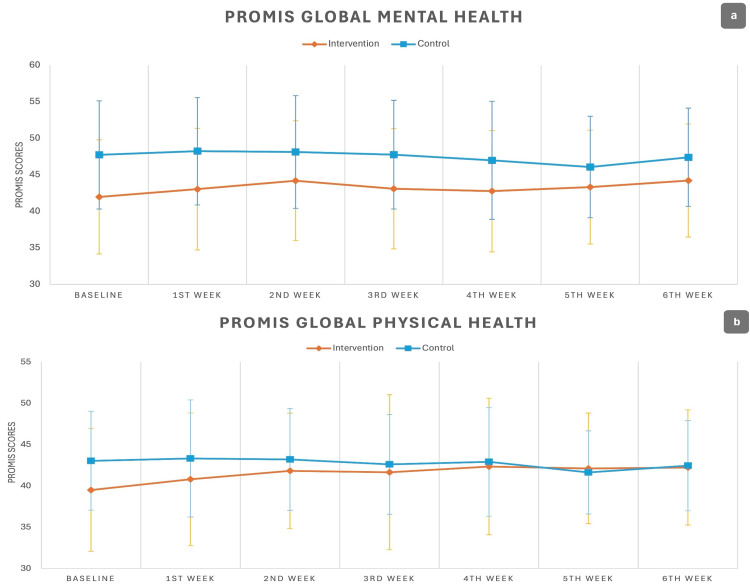
The trend of changes for the PROMIS scores across the study endpoints. (a) PROMIS Global Mental Health: no significant differences were found between the groups. (b) PROMIS Global Physical Health: there was a significant overall difference between the groups (P=0.03) PROMIS: Patient-Reported Outcomes Measurement Information System

The PROMIS Global Physical Health scores showed no significant changes in each endpoint (follow-up visits) compared to the baseline visit (Figure 2). The PROMIS Global Mental Health scores showed significantly higher scores for controls at baseline (47.30±7.53 vs 42.04±7.50; P=0.01); however, the overall changes throughout the study were insignificant (P=0.16) (Figure 2).

## Discussion

In this randomized controlled trial evaluating the efficacy of a home-based seated elliptical training program for CLBP, we observed significant improvements in multiple patient-reported outcomes. The intervention group demonstrated a significant reduction in pain intensity beginning at week 1, with sustained improvements throughout the study period. Pain-related functional interference similarly showed favorable trends in the intervention group, with improvements evident from the first week. While the control group exhibited some improvement in pain interference during week 2, these benefits were not sustained at subsequent time points.

While many studies have emphasized the effect of exercise therapy on LBP, to our knowledge, this study was one of the few to evaluate seated exercise, in our case, seated elliptical devices, as a therapeutic option for CLBP. The observed improvements, despite a limited follow-up period, indicated that integrating this form of exercise may offer meaningful benefits in managing CLBP, as both a preventive and therapeutic method. Prior studies have reported positive effects of elliptical training in similar populations; one study documented a six-week elliptical regimen, performed three times weekly, significantly reducing pain and disability, with a 21% decrease in Oswestry Disability Index (ODI) scores and a 32% reduction in PROMIS-29 pain scores at week 4 [9]. Additionally, a Cochrane systematic review in 2000 synthesizing findings from 39 trials concluded that while exercise has a limited impact on acute LBP, it can facilitate improved function in CLBP [10]. More recent evidence, such as a 2010 systematic review of 37 randomized controlled trials, confirmed that exercise therapy can reduce pain and improve function in CLBP [11]. A 2021 Cochrane review of 249 trials on CLBP exercise interventions found moderate evidence that exercise outperformed standard care or placebo for reducing pain. However, its effect on functional limitations was generally smaller and not clinically significant [4]. Our study contributes to the field by evaluating a unique, accessible exercise modality that may offer distinct advantages, especially for individuals who inevitably have to abide by a sedentary lifestyle or are not able to perform standing exercises for various reasons.

Research indicates that muscle degeneration, including atrophy and increased fat infiltration, is common in the lumbar muscles of individuals with CLBP, particularly affecting stabilizing muscles such as the multifidus and erector spinae [12-14]. Core and back muscle strengthening exercises have been extensively highlighted in the literature as critical for improving clinical outcomes in CLBP patients [15-17]. Additionally, Meng and Yue demonstrated in their meta-analysis, which included eight clinical studies and 310 patients, that aerobic exercise can also provide significant symptomatic relief, as measured by the ODI (standardized mean difference: 1.35; 95% CI: 0.34-2.37; P=0.01) [18]. Complementary findings from Botter et al. showed that sitting ellipticals and active standing workstations can increase heart rate and strengthen back muscles, providing a possible role for these modalities in the treatment of CLBP based on the aforementioned treatment principles [19]. Building on this, our study specifically evaluated sitting ellipticals and demonstrated clinical improvements in pain among CLBP patients. While these results are encouraging, further large-scale and longitudinal studies are needed to fully assess the long-term efficacy of this intervention.

Sedentary behavior including prolonged sitting is a well-established risk factor for CLBP which is linked to the weakening of the core and lower body structures critical for lumbopelvic stability and load management [20,21]. Home-based or workplace-based exercise regimens have shown promise as an accessible intervention to counteract these effects, especially for patients who may have limited access to traditional supervised care. Studies on these interventions consistently demonstrate reductions in both pain intensity and functional limitations, likely owing to the ease with which patients can engage in structured, low-impact physical activity from home [22]. Such programs allow for individualized progression, targeting specific muscular deficits and promoting better adherence, which is essential for long-term outcomes in pain management and functional recovery [23]. By reducing sedentary behavior via adaptable, home-based, or workplace-based training, physicians may offer patients with CLBP a practical and effective approach to improve their condition, particularly for those facing barriers to in-person exercise therapy.

This study offers valuable insights, though certain limitations should be acknowledged. The study population is female-dominant, providing important findings but suggesting the need for future research to include more diverse cohorts to enhance generalizability. The study team concluded the trial after enrolling 54 participants due to logistical constraints, which resulted in a reduction of the study's overall statistical power. CLBP is a non-specific diagnosis, and subgroups with distinct pathologies (e.g., radiculopathy, degenerative disc disease, or facet arthropathy) may respond differently to this intervention. Given the flexion-based nature of the exercise, this therapy may be more appropriate for individuals with extension-based LBP, such as those with facet arthropathy. The absence of supervised training sessions may have influenced adherence and the effectiveness of the exercise regimen; however, this real-world setting reflects the feasibility of the intervention in typical clinical scenarios. Furthermore, the absence of blinding introduces a high risk of performance and detection bias, and the absence of established minimal clinical significance thresholds for the study outcomes limits our ability to interpret the magnitude of the observed effects. Lastly, a longer follow-up period could provide more insight into the outcomes of our training method; however, it was not possible due to financial limitations.

## Conclusions

Our exploratory randomized controlled trial provides preliminary indications that a home-based seated elliptical exercise might improve patient-reported outcomes in individuals with CLBP. While the power of the study was limited by low sample size, the intervention group showed statistically significant improvements in pain intensity and pain-related functional interference compared to the control group. Despite these promising observations, the study participants didn't report statistically significant improvements in global health measures. Our study suggested that simple and accessible exercise devices that can be used by individuals in places where they spend the majority of their daytime sitting or in a sedentary status might help improve CLBP. Future studies should confirm these results in larger, more diverse populations and explore digital methods to monitor adherence and establish a minimal clinically important difference, aiming to strengthen the role of accessible, home-based, or workplace-based exercise in clinical practice.
